# Combination treatment of rituximab and donor platelets infusion to reduce donor‐specific anti‐HLA antibodies for stem cells engraftment in haploidentical transplantation

**DOI:** 10.1002/jcla.23261

**Published:** 2020-02-28

**Authors:** Rongli Zhang, Yi He, Donglin Yang, Erlie Jiang, Qiaoling Ma, Aiming Pang, Weihua Zhai, Jialin Wei, Sizhou Feng, Mingzhe Han

**Affiliations:** ^1^ Transplant Center Institute of Hematology and Blood Diseases Hospital Chinese Academy of Medical Sciences (CAMS) and Peking Union Medical College (PUMC) Tianjin China

**Keywords:** donor platelets, donor‐specific anti‐HLA antibodies, engraftment, haploidentical hematopoietic stem cell transplantation, rituximab

## Abstract

**Background:**

Donor‐specific anti‐human leukocyte antigen (HLA) antibodies (DSAs) in recipients is a risk factor for donor stem cell graft failure in haploidentical hematopoietic stem cell transplantation (haplo‐HSCT), and the treatment to reduce the levels of DSAs is not unanimous. This study was to analysis the role of DSAs for stem cell engraftment and to discuss the effective treatment to reduce DSAs in haplo‐HSCT.

**Methods:**

We retrospectively evaluated the levels of DSAs and the effect of the combination treatment of rituximab and donor platelets (PLTs) for donor stem cell engraftment in haplo‐HSCT patients from June 2016 to March 2018 at our center.

**Results:**

Nine patients (11.5%) out of the total 78 patients were DSAs‐positive and multivariate analysis revealed DSAs was the only factor that affected engraftment. Seven out of the 9 DSAs (+) patients received therapy: Four had antibodies against donor HLA class I (HLA‐I) antigens and were administered two therapeutic amounts of donor apheresis platelets (platelet count approximately 3‐5 × 10^11^) before donor stem cell infusion and the other three patients received a combination therapy of donor apheresis platelets and rituximab due to the antibodies against both donor HLA‐I antigens and HLA class II (HLA‐II) antigens. All the seven patients achieved donor stem cell engraftment successfully, and the DSAs levels decreased rapidly after transplantation.

**Conclusions:**

DSAs is an important factor affecting engraftment in haplo‐HSCT. Donor platelet transfusion is one simple and effective treatment for HLA‐I DSAs, and a combination therapy should be administered if patients have both HLA‐I and HLA‐II antibodies.

## 1 INTRODUCTION

Allogeneic hematopoietic stem cell transplantation is an effective treatment for hematologic malignancies and bone marrow (BM) failure diseases. However, the probability of finding human leukocyte antigen (HLA) matched sibling donors is <30%, and it is even lower in unrelated donors.^1^ Haploidentical donors have gradually become substitutes for traditional donors in recent years, whereas primary graft failure (PGF) remains a life‐threatening complication after transplantation either due to increased transplant‐related mortality following infectious and hemorrhagic complications or due to early relapse in the absence of a functional graft.^2^, ^3^ HLA mismatch and donor‐specific anti‐HLA antibodies(DSAs) in recipients are critical causes of graft failure and malfunction in solid organ transplantation.^4^, ^5^ Recent researches revealed that DSAs played an equally important role in haploidentical hematopoietic stem cell transplant (haplo‐HSCT).^6^, ^7^, ^8^, ^9^ In haplo‐HSCT, the engraftment rate was much lower in DSAs (+) recipients than that in DSAs (−) recipients (61.9% vs 94.4%), and a higher fluorescence intensity of the DSAs was associated with a higher risk of graft failure and transplant‐related mortality.^10^, ^11^, ^12^ Therefore, DSAs must be considered when choosing a haploidentical donor and if a patient had no other alternative donors, he or she should been administered appropriate treatment to reduce the levels of DSAs for donor stem cell engraftment. The pre‐transplanting treatment of DSAs is not unanimous. Plasmapheresis was often used before conditioning, but the antibody levels usually rebounded when donor stem cells were infused.^10^ Rituximab or proteasome inhibitor could inhibit B lymphocytes from producing new antibodies, but it could not reduce the levels of already existing DSAs. Since HLA class I (HLA‐I) antigens are highly expressed in platelet(PLT), donor PLT infusion might neutralize the recipient's anti‐HLA‐I DSAs and rapidly reduce the DSAs levels without increasing the risk of developing acute graft‐versus‐host disease(aGVHD) or other adverse effect.^13^ In the present study, we had analyzed the impact of DSAs on engraftment and the therapy to reduce the levels of DSAs in haplo‐HSCT patients.

## 2 PATIENTS AND METHODS

### 2.1 Patients

Consecutive patients (N = 78) who received a haplo‐HSCT at our Stem Cell Transplant Center between June 2016 and March 2018 were enrolled in the study. The HLA‐A, HLA‐B, HLA‐C, HLA‐DQB1, and HLA‐DRB1 were sequenced in both directions using SeCore SBT Kits (One Lambda). The products were separated on ABI 3730 Genetic Analyzer (Hitachi High‐Technologies Corporation) and analyzed using uTYPE6.0 analysis software (One Lambda). In the present study, haplo‐HSCT was defined as HSCT from donors who share one of the two HLA haplotypes, regardless of the number of HLA‐mismatched loci in A, B, C, DQ, and DR. The study was approved by the ethics committees of the Institute of Hematology, Chinese Academy of Medical Science & Peking Union Medical College according to the guidelines of the Declaration of Helsinki. All patients provided informed consent before participating in the study.

### 2.2 Measurement of anti‐HLA Ab levels

Serum samples were collected from the patients for screening anti‐HLA antibodies with a LABScreen panel reactive antibody (PRA) Kit (One Lambda). Positive samples were further tested for the specificity of the antibodies against HLA‐I (ie, HLA‐A/B/C) and HLA‐II (ie, HLA‐DR/DQ) antigens using a LABScreen Single Antigen Kit (One Lambda). Fluorescence was measured using a Luminex100 flow analyzer (Luminex), and the data were analyzed using the LABScan 100 software (One Lambda). The median fluorescence intensity (MFI) of the PRA beads’ reactions was obtained from the output file generated by the flow analyzer, adjusted for the background signal using the formula: sample beads −negative control beads. The fluorescence intensity of the negative and positive control beads was <100 and >9000, respectively. If the sample data did not fit these conditions, the serum or plasma was treated with ADSORB OUT beads (One Lambda) to reduce background fluorescence. All samples with fluorescence intensity >500 were tested with single Ag beads to confirm and identify sample specificity. MFI was adjusted for the background signal using the abovementioned formula. The samples were considered: negative, MFI < 500; weakly positive, MFI 500‐2000; positive, MFI 2000‐10 000; and strongly positive, MFI > 10 000.

### 2.3 Transplantation protocols and treatment of DSAs

Myeloablative conditioning regimens for patients with hematologic malignancies were as follows: intravenous busulfan (Bu 9.6 mg/kg) or total body irradiation (10 Gy separated by 3 days); cyclophosphamide (CTX, 80 mg/kg); fludarabine (Flu, 90 mg/m^2^); cytarabine (6 g/m^2^); and rabbit antithymocyte globulin (ATG, 10 mg/kg) or pig ATG (80 mg/kg). In patients with aplastic anemia (AA), the conditioning included CTX 150 mg/kg; Flu 120 mg/m^2^; and rabbit ATG 12.5 mg/kg. Cyclosporin A or tacrolimus plus short‐term methotrexate along with mycophenolate mofetil were used for aGVHD prevention. Donor stem cells (BM stem cells and peripheral blood hematopoietic stem cells or only peripheral blood hematopoietic stem cells) were obtained according to the opinions of the donors (If the donor was afraid of bone marrow collection, only peripheral blood stem cells were collected).

For patients who had DSAs against both donor HLA‐I antigens and HLA‐II antigens, one dose of rituximab (375 mg/m^2^) was additionally used 1‐2 weeks before the conditioning(14‐21 days before donor stem cell infusion) combined with two therapeutic amounts of donor apheresis PLT infusion on the day −1 (1 day before donor stem cell infusion). For patients who had DSAs against donor HLA‐I antigens without antibodies against donor HLA‐II antigens, only two therapeutic amounts of donor apheresis PLT infusion were administered on day −1 to reduce the levels of DSAs.

### 2.4 Hematopoietic reconstitution and chimerism analysis

Neutrophil recovery was defined as an absolute neutrophil count (ANC) >0.5 × 10^9^/L for 3 consecutive days, and PLT recovery was defined as PLT count >20 × 10^9^/L for 7 consecutive days without platelet infusion. Graft failure was defined as no appearance or the complete loss of donor‐derived neutrophils by day 28 using short tandem repeat (STR) chimerism analysis. BM puncture was performed on 14, 28, 42, and 60 days after donor stem cell infusion. Chimerism analysis involved the quantitative polymerase chain reaction (PCR) of informative STRs in the recipient and donor. DNA was amplified with fluorescent PCR primers for markers that would distinguish the donor and recipient alleles. Fluorescent PCR products were separated using an Applied Biosystems 3730 Genetic Analyzer (Applied Biosystems), and the Genemapper software (Applied Biosystems) was used to correlate allele peak areas with the percentage of donor or recipient DNA. It was defined as complete donor chimeras when the result of STR was ≥97%. The ratio of X/Y chromosomes was tested using fluorescence in situ hybridization if the gender of donor and recipient was not matched.

### 2.5 Statistical analysis

SPSS 21.0 was used for data processing and analysis. Fisher's exact test was used to compare the differences of engraftment rate among groups, whereas multivariate analysis was performed with logistic regression analysis to identify risk factors that affect donor cell engraftment. Statistical significance was considered when *P* < .05.

## 3 RESULTS

### 3.1 Patient characteristics and donor stem cell engraftment

Overall, 78 patients who underwent haplo‐HSCT from June 2016 to March 2018 were included in this study, and 69 patients were DSAs (−) and 9 (11.5%) were DSAs (+). The characteristic of the patients was showed in Table 1. In the 69 DSAs (−) recipients, PGF occurred in three patients and the remaining 66 recipients (95.6%) had achieved successfully donor cells engraftment. The median time for neutrophil and PLT engraftment was 12 days (range, 10‐22 days) and 17 days (range, 10‐210 days), respectively.

**Table 1:** The characteristic of the patients completed haplo‐HSCT

	HLA Ab‐negative and Non‐DSAs (+)	DSAs (+)
No. of patients	69	9
Median age, years(range)	21 (4‐53)	28 (9‐46）
Female (%)	31 (44.9)	5 (55.5%)
Diagnosis		
AML	22	1
ALL	10	0
MDS	15	7
AA	16	1
CML	3	0
CMML	1	0
Others	2	0
Diseases status at transplant Not in remission (%)	35 (50.8)	6 (66.6)
Donor/recipient relationship		
Sibling	14	3
Offspring to mother	7	2
Offspring to father	8	1
Mother to offspring	17	0
Father to offspring	20	3
cousins	3	0
Stem cell source		
PBSCT	42	4
BM + PBSCT	27	5
Median number of MNC (*10^8^/kg), (range)	8.65 (6.89‐17.2)	4.94 (2.42‐7.79)
Median number of CD34+ (*10^6^/kg), (range)	3.61 (2.02‐11.03)	9.09 (8‐17.11)

At the beginning, the first 4 DSAs (+) patients (Patients 1‐4) underwent routine myeloablative conditioning, which was the same as the other 69 DSAs (−) patients, and no special treatments for DSAs were additional used before donor stem cell infusion. PGF occurred in three patients (Patient 1, Patient 2, and Patient 4). The engraftment rate of the 4 DSAs (+) patients (1/4, 25.0%) was much lower than that of DSAs (−) recipients (66/69, 95.6%; *P* = .001). Multivariate analysis revealed that DSAs was the only factor that affected engraftment (odds ratio [OR] = 34.0; 95% confidence interval [CI], 2.648‐436.545; *P* = .007). The age, HLA mismatch, gender of the donors, ABO mismatch, disease status, stem cell source and amount were all irrelevant to PGF(Tables 2 and 3).

**Table 2:** Identification of risk factors for PGF by univariate analysis

Factors	Value (n = 73)	*P *value
Age, yr, median (range)	24 (4 ~ 59)	.861
Sex, n (%)		
Male	40 (55)	.542
Female	33 (45)	
Diagnosis, n (%)		
AML	20 (27)	.173
ALL	13 (18)	
AA	17 (23)	
MDS	18 (25)	
CML	3 (4)	
CMML	2 (3)	
Diseases status at transplant, n (%)		
Not in remission	41(56)	.452
CR1/2	32 (44)	
Donor/recipient relationship, n (%)		
Sibling	14 (19)	.360
Offspring to mother	8 (11)	
Offspring to father	8 (11)	
Mother to offspring	7 (10)	
Father to offspring	33 (45)	
Others	3 (4)	
HLA matched, n (%)		
5/10	45 (62)	.392
6/10	14 (19)	
7/10	8 (11)	
8/10	4 (5)	
9/10	2 (3)	
Stem cell source, n (%)		
PBSCT	44 (60)	.392
BM + PBSCT	29 (40)	
MNC,×10^8^/L, median (range)	9.99 (6.89‐25.49)	.992
CD34^+^ cells,×10^6^/L, median (range)	3.8 (1.952‐11.05)	.533
Age of donor, y, median (range)	35 (7 ~ 63)	.374
Blood type of donor to receipt, n (%)		
Matched	41 (56)	.024
Mismatched	32 (44)	
DSA, n (%)		
Positive	4 (5)	.000
Negative	69 (95)	

**Table 3:** Identification of risk factors for PGF by multivariate analysis

Factors	Odds ratio	95%CI	*P* value
Blood type of donor to receipt	‐	‐	.998
DSA Positive	34.0	2.648‐436.545	.007

### 3.2 Characteristics of transplant in DSAs (+) patients and the measures for reducing DSAs

There were nine patients with DSAs (+) in this study including the first four patients (Patients 1‐4) mentioned above and the following five new DSAs (+) patients (Patients 5‐9). The characteristics were summarized in Table 4. Among the first 4 DSAs (+) patients, only Patient 3 had successful donor cell engraftment. Patient 4 did not receive a secondary transplant after engraftment failure. He received blood product transfusion along with other support therapy and 6 months later he died of severe infection. Patient 1 and Patient 2 suffered PGF during their first transplants, and then, they underwent their secondary haplo‐HSCT. Patient 2 had no other alternative donor, and his father was still the donor for his secondary transplant. In Patient 1’s first transplant, the donor was her son and her DSAs were against all the 5 mismatched HLA locus antigen (HLA‐A, HLA‐B, HLA‐C, HLA‐DQ, HLA‐DR) of her son. So in her secondary transplant, her uncle was chosen as the donor because her DSAs were against 3 mismatched HLA locus antigens (HLA‐A, HLA‐B, HLA‐DR; Table 5). Due to intravenous Bu used in their first transplants, TBI (total dose 10 Gy) and CTX (80 mg/kg) were used as the myeloablative conditioning and measures against DSAs were administered for their secondary transplants. The following five new DSAs (+) patients (Patients 5‐9) received routine myeloablative conditioning with measures against DSAs directly.

**Table 4:** The characteristic of DSA (+) patients and stem cell engraftment of haplo‐HSCT in their first transplantation

Patient	Gender	Age (y)	Diagnosis/Diseases status	Donor	Donor age (y)	No. of HLA mismatch	Blood type of donor to receipt	Stem cell source	MNC (×10^8^/kg)	CD34^+^ cell (×10^6^/kg)	Treatment for DSAs (Yes/No)	Engraft (Yes/No)
1	F	46	MDS(RAEB‐II)/CR	Son	20	5	A‐A	PB	8.0	3.2	N	N
2	M	26	MDS(RAEB‐I)/NR	Father	53	3	O‐O	PB + BM	7.99	7.66	N	N
3	F	9	MDS(RCC)/NR	Father	34	5	B‐B	PB + BM	10.04	4.94	N	Y
4	M	18	SAA/NR	Father	45	5	B‐B	PB	8.0	2.48	N	N
5	M	28	MDS(RAEB‐II)/CR	Brother	26	3	A‐A	PB + BM	9.96	7.20	Y	Y
6	M	45	MDS(RAEB‐II)/NR	Son	24	5	B‐B	PB + BM	9.09	7.79	Y	Y
7	F	24	AML(MDS)/NR	Brother	21	5	B‐O	PB	8.0	6.4	Y	Y
8	F	36	MDS(RAEB‐I)/NR	Brother	26	3	A‐A	PB + BM	10.9	4.5	Y	Y
9	F	41	AML/CR	Daughter	17	4	B‐B	PB	17.11	2.42	Y	Y

Abbreviations: BM, bone marrow; CR, complete remission; MDS, myelodysplastic syndromes; MNC, mononuclear cell; NR, non‐remission; PB, peripheral blood; RAEB, refractory anemia with excess of blasts; RCC, refractory cytopenia of childhood; SAA, severe aplastic anemia.

**Table 5:** The changes of the DSAs levels of 7 patients during their haplo‐HSCT

Patient	DSAs locus	Secondary transplantation	DSAs treatment	Time of ANC engraftment (d)	Time of PLT engraftment (d)	Before preconditioning	DSAs level（MFI）			
							Before donor PLT infusion	Before stem cell infusion(after donor PLT infusion)	1 mo after stem cell infusion	2 mo after stem cell infusion
1	HLA‐A: 1101； HLA‐B: 5102； HLA‐DRB1:0802	yes	rituximab, donor PLT infusion	+21	+48	HLA‐A:1101：3192.84； HLA‐B:5102：17 688.29； HLA‐DRB1: 0802：11 128.43	^c^	HLA‐A:1101：432.38； HLA‐B:5102：16 226.53； HLA‐DRB1: 0802：8108.77	HLA‐A:1101 (−)； HLA‐B:5102：3303.41； HLA‐DRB1 (−)	HLA‐A:1101 (−)； HLA‐B:5102：588.54； HLA‐DRB1 (−)
2	HLA‐A：0201	yes	donor PLT infusion	+18	^a^	HLA‐A：0201:1478.23	^c^	^c^	(−)	^c^
5	HLA‐A：0201； HLA‐B：5201	no	donor PLT infusion	+19	+31	HLA‐A:0201：14 872.94； HLA‐B:5201：2726.34	^c^	HLA‐A:0201：11 281.45； HLA‐B:5201：593.55	HLA‐A:0201：2509.72； HLA‐B:5201 (−)	HLA‐A:0201 (−)； HLA‐B:5201 (−)
6	HLA‐A：0101	no	donor PLT infusion	+21	+85	HLA‐A：0101:1643.37	^c^	^c^	(−)	^c^
7	HLA‐C：0304 HLA‐DRB1:0803	no	rituximab, donor PLT infusion	+14	^b^	HLA‐C：0304:14 097.2 HLA‐DRB1:0803:979.42	HLA‐C：0304:14 212.5 HLA‐DRB1:0803:^c^	HLA‐C：0304:10 231.49 HLA‐DRB1:0803:^c^	^c^	^c^
8	HLA‐A：2402；HLA‐B: 5101	no	donor PLT infusion	+13	+27	HLA‐A:2402：5770.62 HLA‐B:5101: 2325.23	HLA‐A:2402：5944.84 HLA‐B:5101:762.13	HLA‐A:2402：2731.85 HLA‐B:5101:123.83	HLA‐A:2402： (−) HLA‐B:5101:(‐)	HLA‐A:2402： (−) HLA‐B:5101:(‐)
9	HLA‐B：0801； HLA‐C：0702 HLA‐DRB1: 1501	no	rituximab, donor PLT infusion	+12	+12	HLA‐B:0801；802.81 HLA‐C:0702:10 973.49 HLA‐DRB1:1501:2879.83	HLA‐B:0801；872.71 HLA‐C:0702:10 417.47 HLA‐DRB1:1501:1691.16	HLA‐B:0801；574.85 HLA‐C:0702:10 542.48 HLA‐DRB1:1501:2329.83	HLA‐B:0801； (−) HLA‐C:0702: (−) HLA‐DRB1:1501: (−)	^c^

Abbreviation: MFI, mean fluorescence intensity.

^a^ Patient 2 was not out of PLT infusion and died of severe pneumonia 2 mo after transplant.

^b^ Patient 7 died of severe pneumonia at +18 d after transplant.

^c^ No data.

In this study, total seven patients received measures against DSAs. Three patients, (Patient 1, Patient 7, and Patient 9) had DSAs against donor HLA‐I and HLA‐II antigens, so combination treatments were used: one dose of rituximab (375 mg/m^2^) was additionally used 1‐2 week before conditioning (14‐21 days before donor stem cell infusion) and two therapeutic amounts of donor apheresis PLTs (PLT count was approximately 3‐5 × 10^11^) were infused on −1 day (Table 5). Patient 1 and Patient 9 had successful donor cell engraftment and achieved long‐term survival. The ANC of Patient 7 was >0.5 × 10^9^/L at day + 14 after transplant, and the STR test revealed the donor chimera rate was 99.4%. Unfortunately, Patient 7 died of severe pneumonia and respiratory failure at day + 18. Four patients (Patients 2, 5, 6, and 8) had antibodies against donor HLA‐I antigens without antibodies against to donor HLA‐II antigens. Only two therapeutic amounts of donor apheresis PLT infusion were additionally adopted at day −1 to reduce the levels of DSAs for the 4 patients, and all of them obtained donor cell engraftment (Table 5). Acute GVHD and cytomegalovirus viremia occurred in Patient 2, and he died of severe pneumonia 2 months later after his second transplant.

### 3.3 DSAs levels of patients during transplantation

The median fluorescence intensity (MFI) was used semiquantitatively for estimation of levels of DSAs and for predicting crossmatch results and assessing immunological risk. Crossmatch assays, the patient's serum was incubated with the donor's T and B lymphocytes to assess antibody reactivity directly, were not done in this study. The DSAs levels of Patient 1 and Patient 2 in their secondary transplants and Patients 5‐9 in their primary transplants were shown in Table 5. All the seven patients had antibodies against donor HLA‐I antigens and the levels of DSAs (MFI) were more than 10 000 in four patents (Patients 1, 5,7, and 9), more than 5000 in Patient 8 and weakly positive in Patient 2 and Patient 6. At the same time, three patients (Patient 1, Patient 7, and Patient 9) also had antibodies against donor HLA‐II antigens, MFI was more than 10 000 in Patient 1, MFI was 2879.83 in Patient 9 and weakly positive (979.42) in Patient 7. The DSAs levels against each HLA locus antigen showed different degrees of reduction before stem cell infusion and then decreased rapidly 1 month after stem cell infusion and became negative 1‐2 months after transplant (Table 5).

## 4 DISCUSSION

Anasetti^14^ earlier proposed that PGF was associated with HLA‐mismatched HSCT. The graft failure rate was much higher in patients who possessed anti‐donor lymphocyte antibodies than in patients who did not have these antibodies (39% vs 10%). Some research centers also reported the effect of DSAs on engraftment in allogeneic HSCT recently.^6^, ^8^, ^10^ In our study, the premier four patients (Patients 1‐4) of the 9 DSAs (+) recipients did not take any measures before haplo‐HSCT and then PGF occurred in three patients. The failure rate was much higher than that in DSAs (−) patients. Multiple‐factor analysis revealed that DSAs was the important factor that affected haploid‐matched stem cell engraftment and showed no statistical difference in HLA mismatch, amount of infused donor stem cells, ABO mismatch, killer‐cell immunoglobulin‐like receptors, and primary disease status between engraftment group and graft failure group, which was consistent with Ciurea's research.^15^

It was difficult to speculate the exact DSAs levels due to the limited DSAs (+) patients in our study. In Chang^12^ `s study, DSAs‐positive haploid‐matched recipients were subdivided into three groups according to DSAs MFI: MFI < 2000, MFI: 2000 to <10 000, and MFI: ≥10 000. The primary engraftment failure rate in the three groups was 3.2%, 31.6%, and 60.0%, respectively. As a result, if the patient has DSAs against to a donor's HLA antigens, the donor should be substituted by another one that the patient had no DSAs to the new donor's HLA antigens. In the absence of an alternative suitable donor, it is recommended that measures against DSAs should be taken to reduce the risk of PGF.^7^, ^16^

Methods used to decrease the total antibody levels were reported as follows: plasmapheresis or immunoabsorption for antibody removal, rituximab or proteasome inhibitor for inhibition of antibody production, intravenous immunoglobulin (IVIg) or donor HLA antigens simulant for antibody neutralization, and monoclonal antibodies for inhibition of complement cascade.^10^, ^15^, ^16^, ^17^, ^18^, ^19^ The pre‐transplant treatment of DSAs was not unanimous, and combination therapy was usually adopted.

In our study, total seven patients (two patients for their secondary haplo‐HSCT and five patients for their first haplo‐HSCT) received therapy to lower DSAs levels during transplants and they all obtained complete donor cell engraftments. Patient 1, Patient 7, and Patient 9 had both anti‐HLA‐I and anti‐HLA‐II DSAs, so Rituximab was administered once 1‐2 weeks before the conditioning regimen to inhibit B lymphocytes producing new antibody and donor PLTs used as donor HLA‐I antigens simulant was infused at −1 day to neutralize existing DSAs before donor stem cell infusion. Patients 2, Patient 5, Patient 6, and Patient 8 possessed DSAs against donor HLA‐I antigens; hence, only donor PLT infusions was adopted to lower anti‐HLA‐I DSAs. The results showed that the DSAs levels decreased to a very low level at 1 month and could not be tested at 2 months after transplantation. We speculated that the myeloablative conditioning could also inhibit donor lymphocytes producing new antibodies and donor mononuclear cells infused at 0 day also consumed recipients’ DSAs partly.

Donor PLT infusion was the only special treatment for four patients with anti‐HLA‐I DSAs meanwhile without anti‐HLA‐II DSAs in this study because HLA‐I antigens are highly expressed on PLTs and donor PLTs could act as donor HLA‐I antigens simulant to neutralize the recipients’ anti‐HLA‐I antibodies. The levels of anti‐HLA‐I DSAs decreased rapidly without increasing the risk of developing acute GVHD, and all the four patients obtained complete donor cell engraftments. Other studies showed the similar results.^10^, ^13^, ^20^ Donor PLT infusion might be a simple, effective, and safe treatment for anti‐HLA‐I DSAs. But it is important to note that PLTs do not express HLA‐II antigens, and DSAs against donor HLA‐II antigens will not be consumed.

In our study, plasmapheresis was not used due to: (a) most of our patients were diagnosed as high‐risk MDS without remission and the peripheral blood platelet counts were very low before conditioning, the risk of serious bleeding would be increased during or after plasmapheresis; (b) the day before donor stem cell infusion such as −2 or −3 days was preferred for plasma exchange but there were two risk factors: Bleeding because of lower count of blood platelets after conditioning and ATG concentration decreased.

Most of the DSAs (+) patients in our research were diagnosed as MDS, and they underwent multiple blood products administering before stem cell transplant, which could be one of the causes of producing PRA/DSAs. Ciurea^15^ speculated that multiple pregnancies and multiple transfusions were high‐risk factors for being DSAs positive.

In conclusion, PRA/DSAs is an important factor that may affect donor cell engraftment in haplo‐HSCT. Routine PRA/DSAs testing should be incorporated in donor selection prior to transplantation, and DSAs‐positive donors should be avoided. If there is no other alternative suitable donor, it is recommended that appropriate therapy should be taken to improve donor stem cell engraftment. Donor PLT transfusion is a simple and effective treatment to neutralize anti‐HLA‐I antibodies without the risk of increasing GVHD and other adverse effect, and a combination treatment should be administered if the patient has both anti‐HLA‐I and anti‐HLA‐II antibodies.
